# ESTIMATING THE EFFECTS OF A POTENTIAL VACCINE FOR CYTOMEGALOVIRUS ON DISABILITIES AMONG OLDER ADULTS IN THE US

**DOI:** 10.1093/geroni/igad104.1008

**Published:** 2023-12-21

**Authors:** Yuelin He, Jessica Faul, Kate Duchowny, Chihua Li, Rebecca Stebbins, Grace Noppert

**Affiliations:** University of Michigan Institute for Social Research, Ann Arbor, Michigan, United States; University of Michigan, Ann Arbor, Michigan, United States; University of Michigan, Ann Arbor, Michigan, United States; University of Michigan Institute for Social Research, Ann Arbor, Michigan, United States; King’s College London, London, England, United Kingdom; University of Michigan, Ann Arbor, Michigan, United States

## Abstract

Cytomegalovirus (CMV) infection has been identified as contributing to the development several aging-related diseases, such as disabilities. However, the development of CMV vaccines has been slow, particularly of those targeting older individuals. With few studies that have investigated the potential effects of a CMV vaccine on disabilities among older adults, we aim to study such effects on mobility disabilities by tasks. We use longitudinal data from the Health and Retirement Study (HRS) from 2016 to 2020 to quantify what proportion of disabilities could be prevented in the United States with a viable CMV vaccine. The analytic sample includes N=5,855 individuals aged 65 or above. Under the assumption that a hypothetical vaccine would shift CMV levels from seropositive to seronegative, we construct a binary indicator based on the continuous IgG antibody measure of serum CMV. Using a novel logistic regression-based approach that allows adjustment for continuous age, we estimate attributable fractions (AF) to seropositive CMV levels for five dichotomous outcomes about difficulty with activities of daily living (ADLs), including walking across a room, dressing, bathing, eating, getting in and out of bed, and using the toilet, and stratify the analyses by sex and race/ethnicity. The findings will implicate the potential impact of a vaccine for CMV on mobility disability in older adults, laying the groundwork for future studies to examine the impact of vaccines on disabilities at different time points with varying efficacy levels among the elderly.

